# Refuse Hemmorrhage from the Deep Epigastric Artery, Ten Days after an Appendix Operation through the Rectus Muscle

**Published:** 1913-11

**Authors:** Gaston Torrance

**Affiliations:** Birmingham, Ala.


### PROFUSE HEMMORRHAGE FROM THE DEEP EPIGASTRIC ARTERY, TEN DAYS AFTER AN APPENDIX OPERATION THROUGH THE RECTUS MUSCLE.

By Gaston Torrance, M. D., Birmingham, Ala.

A muscular miner was operated for a gangrenous appendix a week after the initial attack; an inch and a half incision was made through the rectus muscle, the wound was partially closed through and through silk worm gut sutures, using two strands to the suture, one being placed below and two above the drain.

He was placed in a modified Fowler position and was making a rapid and uncomplicated recovery when the hemorrhage occurred; the bleeding was so profuse that the whole bed was saturated and there was a considerable pool on the floor.

The hospital phoned me immediately and having recently seen Dr. Coffee's article, I ordered the wound tightly packed in the lower angle and found the hemorrhage under control when I reached the patient a few minutes later. In order to satisfy myself of the origin of the bleeding, I forcibly wiped the clots away and started up the bleeding again from the deep epigastric vessel.

Two strands of silk worm gut were threaded on a needle and passed through the muscle about an inch below the bleeding point and out again and tied on the skin.

There was never any more trouble and he made a perfect recovery.

